# Anti-cardiolipin IgG autoantibodies associate with circulating extracellular DNA in severe COVID-19

**DOI:** 10.1038/s41598-022-15969-y

**Published:** 2022-07-22

**Authors:** Daniel Bertin, Alexandre Brodovitch, Alexandre Lopez, Robin Arcani, Grace M. Thomas, Abdou Beziane, Samuel Weber, Benjamin Babacci, Xavier Heim, Louise Rey, Marc Leone, Jean Louis Mege, Nathalie Bardin

**Affiliations:** 1 Service d’Immunologie, Biogénopôle, Hôpital de la Timone, Assistance Publique-Hôpitaux de Marseille (AP-HM), Marseille, France; 2 Department of Anesthesiology and Intensive Care, Assistance Publique Hôpitaux de Marseille, Hôpital Nord, Aix-Marseille University, 13015 Marseille, France; 3 Department of Internal Medicine and Clinical Immunology, CHU La Conception, Assistance Publique-Hôpitaux de Marseille (AP-HM), Marseille, France; 4 Aix Marseille Univ, INSERM, INRAE, C2VN, Marseille, France; 5 Aix-Marseille Univ, IRD, MEPHI, IHU-Méditerranée Infection, Marseille, France

**Keywords:** Immunology, Biomarkers, Diseases, Pathogenesis, Risk factors

## Abstract

Whereas the detection of antiphospholipid autoantibodies (aPL) in COVID-19 is of increasing interest, their role is still unclear. We analyzed a large aPL panel in 157 patients with COVID-19 according to the disease severity. We also investigated a potential association between aPL and extracellular DNA (exDNA, n = 85) or circulating markers of neutrophil extracellular traps (NET) such as citrullinated histones H3 (CitH3, n = 49). A total of 157 sera of patients infected by SARS-CoV-2 were collected. A large aPL panel including lupus anticoagulant, anti-cardiolipin and anti-beta-2 glycoprotein I (IgG, IgM and IgA), anti-phosphatidylethanolamine IgA, anti-prothrombin (IgG and IgM) was retrospectively analyzed according to the disease severity. We found a total aPL prevalence of 54.8% with almost half of the cases having aCL IgG. Within an extended panel of aPL, only aCL IgG were associated with COVID-19 severity. Additionally, severe patients displayed higher CitH3 levels than mild patients. Interestingly, we highlighted a significant association between the levels of aCL IgG and exDNA only in aCL positive patients with severe disease. In conclusion, we showed a significant link between aPL, namely aCL IgG, and circulating exDNA in patients with severe form of COVID-19, that could exacerbate the thrombo-inflammatory state related to disease severity.

## Introduction

Autoimmune diseases such as immune thrombocytopenia, autoimmune hemolytic anemia, antiphospholipid syndrome or Kawasaki-like disease, have been reported in coronavirus disease (COVID-19) even in the absence of preexisting immune-mediated inflammatory diseases^1^. Evidence supports that SARS-CoV-2 induces autoimmunity in patients with COVID-19. Exposure to viral epitopes is an identified cause of autoimmunity, previously advanced in infectious diseases with other coronaviruses^2,3^. Molecular mimicry between SARS-CoV-2 protein and self-antigen has been proposed as an autoimmune trigger in patients genetically prone^4–6^. Cells infected with SARS-CoV-2 can escape type I interferon (IFN) response, leading to an uncontrolled viral replication^7^. The influx of neutrophils and monocytes from the circulation into tissues leads to an increased production of pro-inflammatory cytokines that are known to play a major role in the pathogenesis of autoimmune disease^8^. The cytolytic effect of the virus and its massive replication^9^ contribute to the induction of cell death that will trigger autoimmunity and autoantibodies such as antinuclear autoantibodies (AN)^10^. Interestingly, it has also been recently shown that levels of molecules with Damage Associated Molecular Patterns (DAMPs) such as extracellular DNA (exDNA) increased with COVID-19 severity^11^. Due to persistent release or inefficient clearance, exDNA exhibit a negative effect on body hemostasis by initiating multiple inflammatory signaling pathways^12^. In addition it has been proposed that neutrophils can produce neutrophil extracellular traps (NETs) by releasing decondensed chromatin to trap SARS-CoV-2. Therefore, an excess of exDNA or NETs have been reported as risk factors of severe illness during COVID-19 infection^13^. Importantly, they can contribute to the generation of a thrombo-inflammatory state, as observed in acute respiratory distress syndrome, or in cancer^14,15^.

As 5–15% of patients suffering from COVID-19, particularly the elderly and people with preexisting cardiovascular diseases, develop severe pneumonia and coagulopathy disorders^16,17^, we hypothesized that an autoimmune mechanism would exacerbate the inflammatory response and contribute to excessive exDNA or NET production. In line with this, we recently described the presence of antiphospholipid autoantibodies (aPL) in COVID-19 patients, by showing that anti-cardiolipin IgG autoantibodies (aCL) are highly and independently associated with COVID-19 severity^18^. aPL represent a large family of autoantibodies that are central for the diagnosis of antiphospholipid syndrome (APS), an autoimmune-associated coagulopathy, and are also considered as vascular risk factors^19^. In APS, thrombotic effects have been mainly associated with aPL-activated cells such as endothelial cells or platelets. aPL can stimulate neutrophils to produce exDNA and NETs in APS patients^20^. Similarly, purified IgG fraction isolated from COVID-19 patients with aPL has been shown to promote NETosis when incubated with neutrophils purified from healthy subjects^21^. Several studies reported the presence of aPL in COVID-19 patients, but conclusions should be clarified about their frequency, type and clinical effects. To this end, we retrospectively assessed in 157 patients with COVID-19 a large aPL panel. It includes lupus anticoagulant (LA), aCL IgG/IgM/IgA, anti-beta-2 glycoprotein I (aB2GPI) IgG/IgM/IgA, anti-phosphatidylethanolamine (aPE) IgG/IgM, and anti-prothrombin (aPT) IgG/IgM autoantibodies. To further investigate the pathological role of aPL produced in COVID-19, we measured circulating exDNA and CitH3 levels, and analyzed them according to aPL detection and disease severity.

## Material and methods

### Patients

All patients with COVID-19 were confirmed to be infected by SARS-CoV-2 by real-time reverse transcriptase PCR (RT-qPCR) testing or by a positive serology for anti-SARS CoV-2 IgG.

aPL dosage was performed on serum samples collected from 157 COVID-19 patients admitted between March 2020 and July 2021 in the University Hospital of Marseilles (France) and consecutively referred to the hospital laboratory for immunological exploration.

Clinical data were collected from the day of sampling. They included co-morbidities (history of thrombosis, history of stroke, coronary heart disease, hypertension, diabetes, heart failure and chronic respiratory disease), clinical presentation (mild or severe), and duration of symptoms.

### Definitions

“Severe clinical presentation” was defined based on at least one of the following criteria: respiratory rate > 30 cycles/min, oxygen saturation ≤ 93%, PaO_2_/FiO_2_ ratio ≤ 300 mmHg, shock (defined by the need of vasopressors) or respiratory failure requiring the admission to intensive care units (ICUs)^22^. “Thrombosis” was defined as the formation of a thrombus within a blood vessel (artery or vein) confirmed by ultrasound, magnetic resonance imaging (MRI) or X-ray Computed Tomography (CT). “Chronic respiratory disease” included chronic obstructive pulmonary disease, asthma, or lung cancer. “Heart failure” included class III or class IV stages according NYHA classification. “Acute respiratory distress syndrome” was defined according to Berlin definition^23^.

### Treatments

The patients received anticoagulant treatment with prophylactic heparin consisting of subcutaneous low molecular weight heparin (enoxaparin 4000 IU/day) or subcutaneous unfractionated heparin (5000 IU every 12 h) if patients had a clearance < 30 mL/min or if admitted to ICU. Combination of non-invasive ventilation/high flow oxygen or mechanical ventilation were used when respiratory support was needed.

### ICU control group

To assess the specificity of aPL in COVID-19, patients admitted to ICU with a negative SARS-CoV-2 RT-qPCR and/or absence of anti-SARS CoV-2 IgG, and screened for aPL in ICU during the study period, were analyzed according to their etiology of ICU hospitalization.

### Ethics

All serum samples collected from patients infected by SARS-CoV-2 were part of a declared Biobank (DC 2020-4028) in compliance with ethical directives. For ICU non-COVID-19 patients, samples were part of a declared Biobank (DC 2012-1704) in compliance with ethical directives. Informed consent was obtained from all participants. This study received approval from the national review board Comité de Protection des Personnes Ile de France XI (20027-60604, March 25th 2020) and fulfilled local requirements in terms of data collection and protection of data (RGPD/APHM 2020-80). This study was conducted according to the Declaration of Helsinki.

### Biological tests

aCL and aB2GPI levels were measured by ELISA with Cardiolisa Theradiag (Marne la Vallée, France) and Orgentec Diagnostica (Mainz, Germany), respectively. Positive cut-off were set-up respectively at 15 U/mL and 8 U/mL according to manufacturers’ recommendations and on-site validation. To avoid non-specific binding issues, each positive sample was duplicated and serum non-specific background of uncoated well was subtracted from the measured optical density (OD) of coated well. To assess cofactor dependence of aCL IgG, all positive sera were tested with another ELISA assay (AIDA, Bad Kreuznach, Germany) with manufacturers cut-off equals to 15 U/mL.

aPT were detected with an in-house ELISA previously described^24^. ΔOD for each sample was calculated by subtracting OD of coated well from OD obtained with non-coated well. The aPT levels were reported as a ratio of ΔOD of patient/ΔOD of a selected control serum with a ΔOD at the cut-off value. The result is positive when the ratio is above 1.

aPE were measured with an in-house ELISA previously described^25^. The optical density (OD) of each well was measured at 405 nm and the OD of non-coated well was subtracted for each sample (ΔOD). These cut-off values were 0.47 and 0.68 ΔOD, corresponding to the following arbitrary units: 18 and 59 U/mL for aPE IgG and aPE IgM, respectively.

Antinuclear autoantibodies (ANA) in patients' sera were detected by an indirect immunofluorescence (IIF) assay (Kallestad HEp-2 Cell Line Substrate, Bio-Rad, Hercules, CA, USA). Anti-double stranded DNA (ds-DNA) and anti-extractable nuclear antigen (ENA) antibody levels were measured in sera with fluorescence-enzyme immunoassay (EliA; Phadia, Uppsala, Sweden).

Quantification of exDNA levels in serum was performed according to manufacturer's instructions using the Quant-iT PicoGreen DNA assay kit (Invitrogen, Thermo Fisher Scientific, Waltham, MA, USA) as previously published^26^.

Quantification of CitH3 levels in serum was performed according to manufacturer's instructions using Citrullinated Histone H3 (Clone 11D3) ELISA Kit (Cayman Chemical, Ann Arbor, MI, USA).

### Statistical analysis

Analysis was performed using R version 3.03 (R Development Core Team) and GraphPad Prism V6.05 (GraphPad Software, La Jolla, CA, USA). Data are described as Mean ± standard deviation in the tables. Shapiro–Wilk test was used to test for data normality and two-tailed Student *t* test was used to test variable differences between groups. Pearson’s Chi-squared test was used to test difference in frequencies between groups for categorical variables. Correlations between markers were evaluated using Pearson correlation analysis. Significance level was set at 0.05. The study was conducted in accordance to the STROBE statement.

## Results

### Characteristics of patients with COVID-19

**Figure 1:**
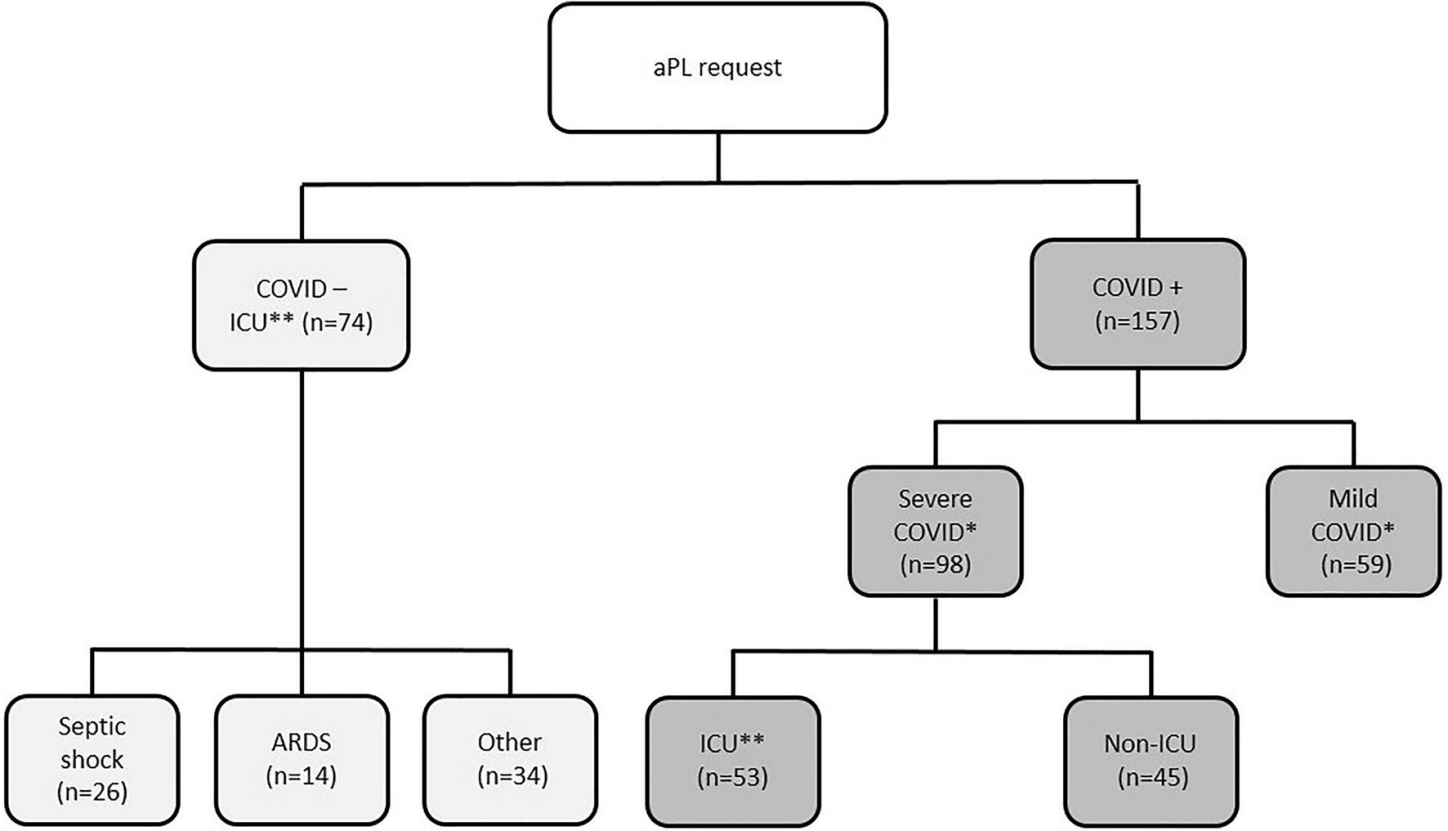
Flow Chart of patients. *ARDS* acute respiratory distress syndrome, *ICU* Intensive Care Unit. Two comparison were performed in this study: one between severe and mild COVID groups (*) and another between COVID ICU and non COVID ICU groups (**).

A total of 157 patients infected by SARS-CoV-2 were included in this study (Fig. 1, Table 1), with 53 hospitalized in ICU. The mean age of patients was 68 ± 16 years and 57% of them were males. According to the clinical presentation at sampling time, this cohort was divided into two groups: mild (n = 59) and severe (n = 98), as defined above.

**Table 1:** Patients with COVID-19 characteristics.

	Mild (n = 59)	Severe (n = 98)	Total (n = 157)	p value
**Age—years**				0.445^1^
Mean (SD)	66.4 (18.6)	68.5 (14.7)	67.7 (16.2)	
Range	20–92	35–96	20–96	
Female—n (%)	26 (44.1%)	42 (42.9%)	68 (43.3%)	0.882^2^
**Duration of symptoms—days**				**< 0.001**^**1**^
Mean (SD)	11.1 (6.0)	16.8 (10.182)	14.7 (9.3)	
Range	1–32	2–47	1–47	
Missing values	1	1	2	
**Medical history—n (%)**				
Thrombosis	9 (15.3%)	8 (8.2%)	17 (10.8%)	0.166^2^
Heart failure	2 (3.4%)	6 (6.1%)	8 (5.1%)	0.451^2^
Stroke	5 (8.5%)	7 (7.1%)	12 (7.6%)	0.761^2^
Coronary heart disease	7 (11.9%)	6 (6.1%)	13 (8.3%)	0.206^2^
Diabetes	14 (23.7%)	19 (19.4%)	33 (21.0%)	0.518^2^
High blood pressure	31 (52.5%)	45 (45.9%)	76 (48.4%)	0.421^2^
Chronic renal failure	6 (10.2%)	6 (6.1%)	12 (7.6%)	0.355^2^
Chronic respiratory disease	7 (11.9%)	8 (8.2%)	15 (9.6%)	0.445^2^
Cancer	9 (15.3%)	17 (17.3%)	26 (16.6%)	0.733^2^
**Anticoag. treatment—n (%)**	31 (53.4%)	77 (78.6%)	108 (69.2%)	**0.001**^**2**^
Missing values	1	0	1	
**Invasive ventilation—n (%)**	0 (0.0%)	35 (35.7%)	35 (22.3%)	**< 0.001**^**2**^
**Thrombotic events—n (%)**	1 (2.6%)	7 (8.3%)	8 (6.6%)	0.239^2^
Missing values	21	14	35	
**PMN (G/L)**				**< 0.001**^**1**^
Mean (SD)	3.943 (1.7)	6.8 (4.0)	5.7 (3.6)	
Range	1.2–11.0	1.0–20.0	1.0–20.0	
Missing values	1	0	1	
**Lymphocytes (G/L)**				0.861^1^
Mean (SD)	1.3 (0.5)	1.3 (0.8)	1.3 (0.7)	
Range	0.4–3.0	0.1–3.9	0.1–3.9	
Missing values	1	0	1	
**NLR**				**< 0.001**^**1**^
Mean (SD)	3.6 (2.3)	8.0 (8.8)	6.4 (7.4)	
Range	0.7–11.2	0.9–60.000	0.7–60.0	
Missing values	1	0	1	
**Eosinopenia—n (%)**	25 (43.1%)	67 (68.4%)	92 (59.0%)	**0.002**^**2**^
Missing values	1	0	1	
**Death—n (%)**	1 (2.6%)	18 (21.7%)	19 (15.7%)	**0.007**^**2**^
Missing values	21	15	36	

*PMN* polymorphonuclear neutrophils, *NLR* neutrophil–lymphocyte ratio, ^1^Student’s *t* test, ^2^Pearson’s Chi-squared test.

No differences in terms of age, gender, and co-morbidities were observed between the two groups. In contrast, the duration of symptoms was longer in the severe group than in the mild group (p < 0.001). The patients in the severe group were more often anticoagulated (p = 0.001) and invasively ventilated (p < 0.001) than those in the mild group. Fatal evolution was significantly associated with severity (p = 0.007).

Regarding biological variables, elevated neutrophil count, neutrophil-to-lymphocyte ratio (NLR), and eosinopenia were significantly associated with the severe form of the disease (p < 0.001, p < 0.001, p = 0.002, respectively).

### Antinuclear autoantibodies

ANA detection by IIF was performed in 105 patients from our cohort. Among them, 74 (70.5%) were tested negative. Of the 31 COVID-19 patients positive for ANA, 15 were in the mild group and 16 in the severe group (p = 0.459) (Table 2). Among these 31 patients, 29 (93.5%) had a speckled fluorescence pattern and 13 out of 29 had a fluorescence titer higher than 320. Autoantibodies against dsDNA or ENA were not detected, except for one patient positive for anti-CENPB autoantibody, in agreement with the fluorescence pattern.

**Table 2:** Antinuclear and antiphospholipid autoantibodies in patients with COVID-19.

	Mild (n = 59)	Severe (n = 98)	Total (n = 157)	p value
**ANA positivity—n (%)**	15 (33.3%)	16 (26.7%)	31 (29.5%)	0.459
Missing values	14	38	52	
**aPL positive—n (%)**	26 (44.1%)	60 (61.2%)	86 (54.8%)	**0.036**
aCL IgG	4 (6.8%)	37 (37.8%)	41 (26.1%)	**< 0.001**
aCL IgM	6 (10.2%)	7 (7.1%)	13 (8.3%)	0.505
aCL IgG IgA	0 (0.0%)	2 (2.1%)	2 (1.3%)	0.263
Missing values	1	4	5	
aB2GPI IgG	2 (3.4%)	4 (4.1%)	6 (3.8%)	0.827
aB2GPI IgM	4 (6.8%)	6 (6.1%)	10 (6.4%)	0.870
aB2GPI IgA	10 (17.2%)	12 (12.8%)	22 (14.5%)	0.446
Missing values	1	4	5	
aPE IgG	7 (11.9%)	18 (19.1%)	25 (16.3%)	0.236
Missing values	0	4	4	
aPE IgM	2 (3.4%)	4 (4.3%)	6 (3.9%)	0.788
Missing values	0	4	4	
aPE IgA	1 (7.7%)	0 (0.0%)	1 (2.3%)	0.124
Missing values	46	68	114	
aPT IgG	0 (0.0%)	1 (1.1%)	1 (0.7%)	0.445
Missing values	8	10	18	
aPT IgM	5 (9.8%)	17 (19.3%)	22 (15.8%)	0.139
Missing values	8	10	18	

*aPL* antiphospholipid, *aCL* anti-cardiolipin, *aB2GPI* anti-beta-2 glycoprotein I, *aPE* anti-phosphatidylethanolamine, *aPT* anti-prothombin autoantibodies, *ANA* antinuclear autoantibodies.

### Antiphospholipid auto-antibodies analysis

As most of our patients were treated with anticoagulants, results of lupus anticoagulant (LA) were interpretable in 21 patients only. Among them, 14 were found negative and 7 positive, 3 of them with a mild and 4 with a severe form of the disease.

**Figure 2:**
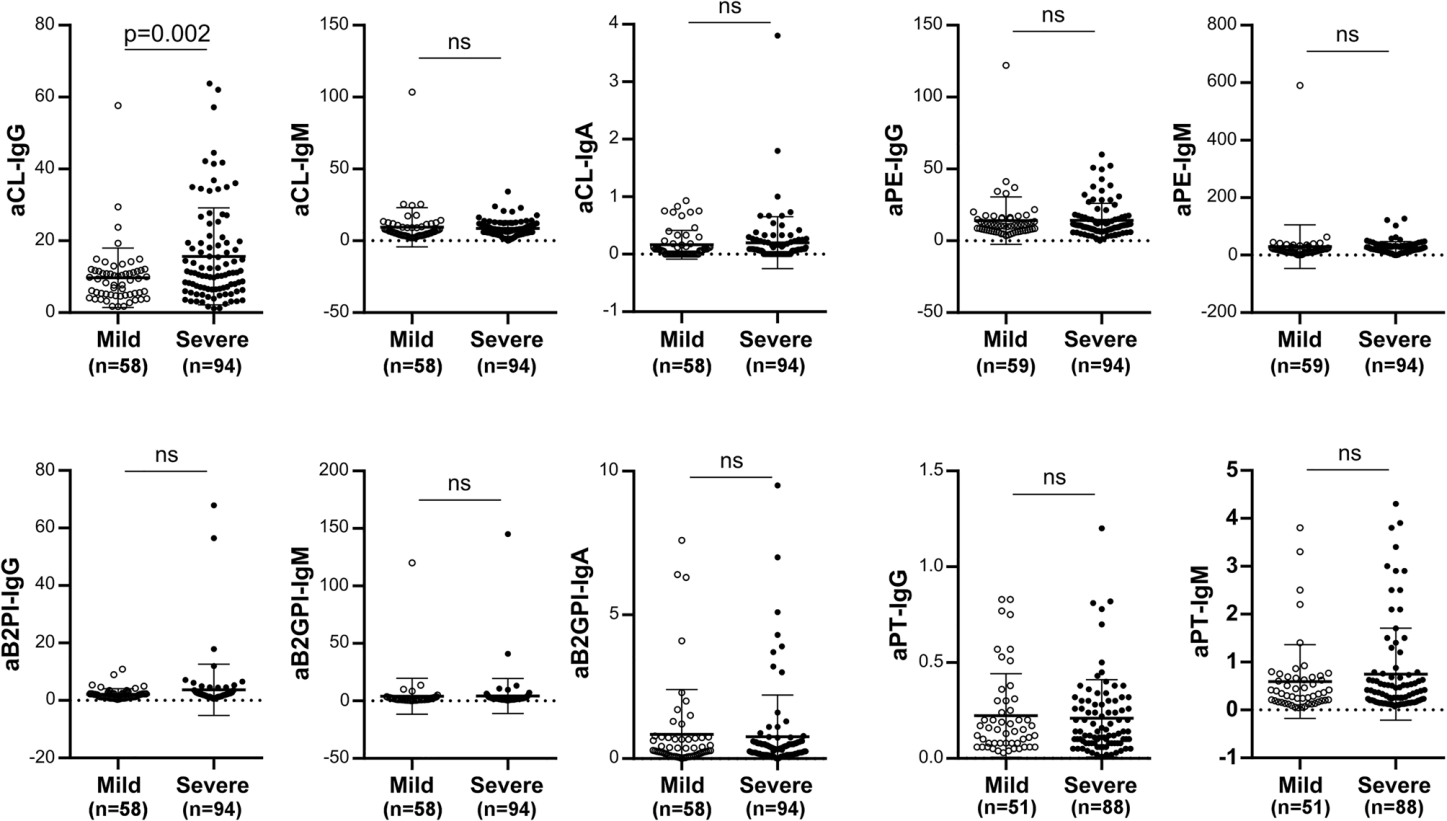
Antiphospholipid autoantibody levels in mild and severe patients with COVID-19. *aCL* anti-cardiolipin, *aB2GPI* anti-beta-2 glycoprotein I, *aPE* anti-phosphatidylethanolamine, *aPT *anti-prothrombin autoantibodies. Statistical analysis performed with Student's *t* test. *ns* not significant.

For all patients, a large panel of aPL was investigated including lupus anticoagulant (LA), aCL IgG/IgM/IgA, aB2GPI IgG/IgM/IgA, aPE, and aPT IgG/ IgM (Table 2). The total prevalence rate was equal to 54.8% for aPL (86/157), and 26.1% for aCL IgG (41/157) positivity. Interestingly, only aCL IgG showed a significantly higher prevalence in the severe group (37.8%, 37/98) than in the mild group (6.8%, 4/59) (p < 0.001). The levels of aCL IgG were significantly higher in the severe group (Fig. 2, Mild: 9.74 ± 8.20 U/mL; Severe: 15.80 ± 13.34 U/mL; p = 0.002). A prevalence above 10% was found for aB2GPI IgA (14.5%, 22/157), aPE IgG (16%, 25/157) and aPT IgM (15.8%, 22/157). However, no association was identified with disease severity.

We aimed to investigate whether aPL positivity was associated with COVID-19 independently of severe conditions such as ARDS and/or septic shock. To this purpose, we conducted an analysis on COVID-19 patients admitted to ICUs, and compared them with patients admitted to ICUs negative for SARS-CoV-2 by RT-qPCR and/or anti-SARS CoV-2 IgG serology. Out of 127 ICU patients screened for aPL during the study period, 29 (22.8%) were found positive for at least one conventional aPL (aCL or aB2GPI IgG or IgM). Of them, 20 patients (69%) developed COVID-19-associated ARDS (17/20 (85%) having aCL IgG), 2 patients (6.9%) suffered from a non-COVID-19-related ARDS and 4 patients (13.8%) with a non-COVID-19 related-septic shock. In the ICU group with aPL positivity (Table 3), characteristics of COVID-19 patients were compared with those of patients without COVID-19, showing that non-COVID-19 patients had a higher frequency of autoimmune diseases than those without COVID-19 (44.4% vs 5%, p = 0.022). In 93 patients negative for aPL, 28 patients (30.1%) were treated for COVID-19-related ARDS, 12 patients (12.9%) for non-COVID-19 related-ARDS and 22 patients (23.7%) for non-COVID-19 related-septic shock. Consequently, COVID-19-related ARDS was significantly associated with aPL positivity (69.0% vs. 30.1%, p < 0.001). There was no association between non-COVID-19-related ARDS and aPL positivity nor between septic shock and aPL positivity (6.9% vs. 12.9%, p = 0.38 and 13.8% vs. 23.7%, p = 0.26, respectively). Finally, aPL positivity was more frequent in COVID-19-related-ARDS than in non-COVID-19 related-septic shock (69% vs. 13.8%, p < 0.0001).

**Table 3:** Characteristics of patients admitted in ICU with aPL positivity.

Characteristics	Non-COVID-19 patients with aPL positivity (n = 9)	COVID-19 patients with aPL positivity (n = 20)	p
Age (mean ± SD)	55.9 ± 14.0	64.1 ± 7.9	0.13
Male gender (n, %)	5 (55.6)	15 (75.0)	0.30
**aPL positivity (n, %)**			
aCL IgG	8 (88.9)	17 (85)	0.78
aCL IgM	1 (11.1)	5 (25)	0.39
aB2GP1 IgG	0	1 (5)	0.49
aB2GP1 IgM	1 (11.1)	2 (10)	0.93
**Level of aPL (U/mL)**			
aCL IgG	30.3 ± 17.2	28.6 ± 16.5	0.81
aCL IgM	10.7 ± 12.1	9.1 ± 6.3	0.72
aB2GP1 IgG	1.8 ± 0.8	2.3 ± 2.8	0.45
aB2GP1 IgM	2.7 ± 3.3	5.2 ± 12.6	0.42
**Comorbidities**			
Autoimmune disease	4 (44.4)	1 (5)	**0.02**
Cardiovascular disease	1 (11.1)	4 (20)	1
Hypertension	2 (22.2)	6 (30)	1
Diabetes	2 (22.2)	6(30)	1
Dyslipidemia	0	6 (30)	0.14
**Evolution in ICU**			
ARDS	2 (22.2)	20 (100)	**< 0.0001**

*aPL* antiphospholipid, *aCL* anti-cardiolipin, *aB2GPI* anti-beta-2 glycoprotein I autoantibodies, *ICU* intensive care unit, *ARDS* acute respiratory distress syndrome, *n* number.

### Anti-cardiolipin IgG characterization and clinical association

Taking into account the methodological issues on ELISA assays and the lack of agreement between methods, we wanted to confirm the aCL positivity of 29 patients. We used an ELISA using beta-2 glycoprotein I as a sole cofactor source (assay 2) and found that 62% of the 29 positive aCL patients were also positive by using assay 2.

To further analyze aCL IgG association with the severe form of COVID-19, we also analyzed aCL IgG with clinical characteristics and biomarkers (Table 4). In addition to the disease severity, a significant association of aCL IgG positivity was found with symptom duration (p < 0.001), transfer to ICU (p < 0.001) and invasive ventilation (p < 0.001). No association was found with in-hospital mortality, clinical history of patients or thrombotic events occurring during the active phase of the disease. Concerning the biomarkers, we observed one significant association between aCL IgG and elevated polymorphonuclear neutrophils (PMN) counts (p = 0.007).

**Table 4:** Anti-cardiolipin autoantibodies in patients with COVID-19.

	aCL IgG Neg (n = 116)	aCL IgG Pos (n = 41)	Total (n = 157)	p value
**Age—years**				0.668^1^
Mean (SD)	68.1 (16.2)	66.8 (16.5)	67.7 (16.2)	
Range	20–96	30–94	20–96	
Female—n (%)	55 (47.4%)	13 (31.7%)	68 (43.3%)	0.081^2^
**Duration of symptoms—days**				**< 0.001**^**1**^
Mean (SD)	13.0 (8.1)	19.5 (10.7)	14.7 (9.3)	
Range	1–47	3–47	1–47	
Missing values	1	1	2	
Severe symptoms—n (%)	61 (52.6%)	37 (90.2%)	98 (62.4%)	**< 0.001**^**2**^
Intensive care—n (%)	27 (23.3%)	26 (63.4%)	53 (33.8%)	**< 0.001**^**2**^
**Medical history—n (%)**				
Thrombosis	13 (11.2%)	4 (9.8%)	17 (10.8%)	0.797^2^
Heart failure	6 (5.2%)	2 (4.9%)	8 (5.1%)	0.941^2^
Stroke	9 (7.8%)	3 (7.3%)	12 (7.6%)	0.927^2^
Coronary heart disease	9 (7.8%)	4 (9.8%)	13 (8.3%)	0.690^2^
Diabetes	23 (19.8%)	10 (24.4%)	33 (21.0%)	0.538^2^
High Blood Pressure	59 (50.9%)	17 (41.5%)	76 (48.4%)	0.301^2^
Chronic renal failure	11 (9.5%)	1 (2.4%)	12 (7.6%)	0.145^2^
Chronic respiratory disease	9 (7.8%)	6 (14.6%)	15 (9.6%)	0.198^2^
Cancer	20 (17.2%)	6 (14.6%)	26 (16.6%)	0.699^2^
**Anticoag. treatment—n (%)**	74 (64.3%)	34 (82.9%)	108 (69.2%)	**0.027**^**2**^
Missing values	1	0	1	
**Invasive ventilation—n (%)**	17 (14.6%)	18 (43.9%)	35 (22.3%)	**< 0.001**^**2**^
**Thrombotic events—n (%)**	5 (5.6%)	3 (9.4%)	8 (6.6%)	0.453^2^
Missing values	26	9	35	
**PMN (G/L)**				**0.007**^**1**^
Mean (SD)	5.3 (3.5)	7.0 (3.6)	5.7 (3.6)	
Range	1–20	1.6–15	1–20	
Missing values	1	0	1	
**Lymphocytes (G/L)**				0.316^1^
Mean (SD)	1.3 (0.7)	1.4 (0.7)	1.3 (0.7)	
Range	0.1–3.9	0.2–3.1	0.1–3.9	
Missing values	1	0	1	
**NLR**				0.588^1^
Mean (SD)	6.2 (7.7)	6.9 (6.7)	6.4 (7.4)	
Range	0.7–60	1.2–37.9	0.7–60	
Missing values	1	0	1	
**Eosinopenia**	66 (57.4%)	26 (63.4%)	92 (59.0%)	0.501^2^
Missing values	1	0	1	
**Death—n (%)**	15 (16.7%)	4 (12.9%)	19 (15.7%)	0.619^2^
Missing values	26	10	36	
**Extracellular DNA (ng/mL)**				**0.031**^**1**^
Mean (SD)	1337.3 (765.5)	1777.7 (988.7)	1461.6 (852.1)	
Range	176.2–2922.0	381.7–3977.1	176.2–3977.1	
**Citrullinated histone H3 (ng/mL)**				0.360^**1**^
Mean (SD)	7.8 (7.7)	10.3 (11.4)	8.5 (9.0)	
Range	0.4–28.3	0.6–34.3	0.4–34.3	
Missing values	82	26	108	

*PMN* polymorphonuclear neutrophils, *NLR* neutrophil–lymphocyte ratio, ^1^Student’s *t* test, ^2^Pearson’s Chi-squared test.

### Anti-cardiolipin IgG and circulating extracellular DNA association

**Figure 3:**
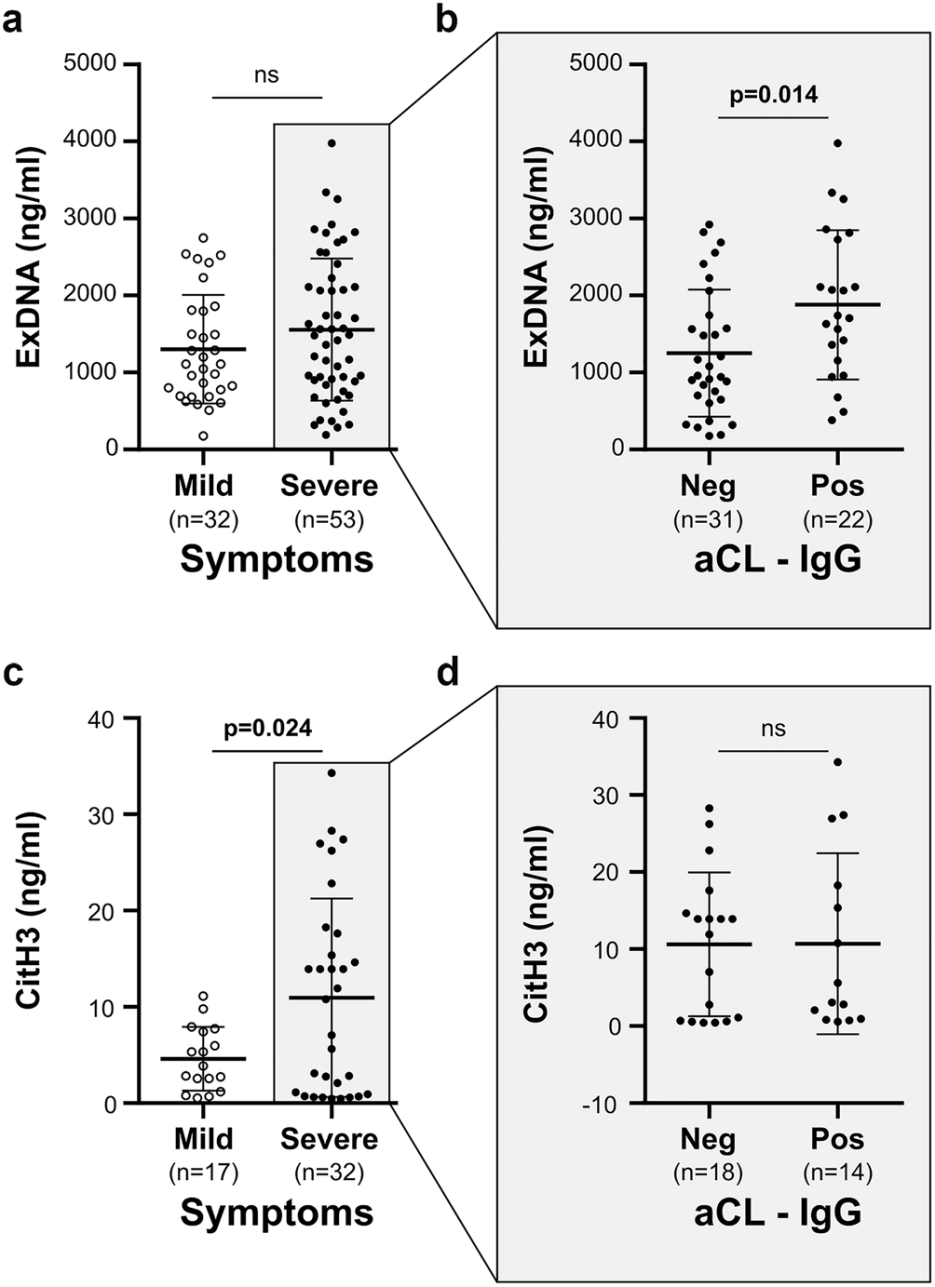
Extracellular DNA and citrullinated histones H3 levels in patients with COVID-19. Extracellular DNA levels in mild/severe COVID-19 patients (**a**) and in aCL IgG positive (Pos)/negative (Neg) patients with severe COVID-19 (**b**). Citrullinated histones H3 levels in mild /severe COVID-19 patients (**c**) and in aCL IgG positive (Pos)/negative (Neg) with severe COVID-19 (**d**). Statistical analysis performed with Student's *t* test. *aCL* anti-cardiolipin, *ns* not significant.

Since exDNA have been recently proposed as markers of COVID-19 complications, we investigated a potential association between aCL and exDNA. From the 85 patients tested, we found a significant association between aCL IgG positivity and exDNA levels in serum (Table 4). Indeed, circulating exDNA levels were significantly higher in aCL IgG positive patients than in aCL IgG negative ones (p = 0.031, Table 4). There was weak correlation between exDNA and aCL IgG levels (Pearson’s r = 0.216, n = 85, p = 0.047) and no association between disease severity and circulating exDNA levels considering the whole population of patients (Fig. 3a). However, among patients with severe COVID-19, exDNA levels were significantly higher (p = 0.014) in aCL IgG positive patients than in IgG aCL negative ones (Fig. 3b).

### Anti-cardiolipin IgG and citrullinated histones H3 association

Because aPL are known to promote NET release from patients with COVID-19, we investigated a potential association between aCL and CitH3, a specific marker of NETs. We measured CitH3 levels on available samples of patients with COVID-19 (n = 49 samples). We found a significant association (p = 0.024) between disease severity and NETs levels (Fig. 3c). Regarding the severe group of patients for which 32 samples were available, no difference was observed in NETs levels according to aCL positivity, in contrast to exDNA levels (Fig. 3d).

## Discussion

Our study showed an association between aPL and circulating exDNA in patients with severe form of COVID-19, that could be involved in the exacerbation of the thrombo-inflammatory response associated with the disease severity.

Several studies reported the presence of aPL in COVID-19, but contradictory results have been found regarding the prevalence, type and clinical relevance of aPL. In our study, we found a total aPL prevalence of 55% with almost half of positive aCL IgG and showed that, within an extended panel of aPL, aCL IgG were associated with severe forms of the disease. This result confirmed our previous data performed on 56 patients with COVID-19 for which only conventional aPL had been sought^18^.

The search for LA was difficult to interpret because the patients affected by COVID-19 received anticoagulant therapy. Devreese et al. found 23% of patients positive for aCL and/or aB2GPI and more than 50% of patients positive for LA^27^, whereas Cristiano et al. described a lower percentage of positivity of around 5% and 2% for aCL and/or aB2GPI and LA, respectively^28^. In accordance with our data, Zuo et al. found a total prevalence of more than 50% in their cohort of 172 patients with COVID-19, with a predominance of aCL^21^. Trahtemberg et al. also corroborated our data by showing that aCL are predominant and associated with disease severity^29^. This heterogeneity of results could be interrelated to the methodology used and/or population studied. Due to the difficult interpretation of aPL positivity, we systematically controlled positive samples in duplicate, and, to address the specificity, the absorbance of uncoated wells treated in the same conditions was systematically subtracted to avoid noise from nonspecific binding. Moreover, in this study, aCL IgG positivity was confirmed by using another ELISA assay and showed that majority of aCL IgG were beta-2‐glycoprotein I cofactor dependent, as described for pathogenic aPL in APS^30^. In addition, to test whether aPL could be generated by ARDS or septic shock, a control cohort of 122 ICU patients, showing that aPL positivity, mostly for aCL, was significantly associated with COVID-19 and not with non-COVID-19 related-ARDS or related-septic shock.

In our study, the presence of aCL was rather associated with inflammation than thrombosis. Indeed, most of patients were on anticoagulant medication, and we did not have controls before the infection. It is therefore difficult to discuss about the association between aCL and thrombosis. Nevertheless, this finding invites to propose a follow-up of patient’s aCL positivity, especially since the persistence of various symptoms in patients who recovered from COVID-19 was recently defined as long COVID or post-covid syndrome (PCS)^31^. In PCS, persistent neurological symptoms have been described, and we can note that neurological disorders are also described in APS^32^.

In contrast to autoantibodies directed against cardiolipin, no association between ANA and disease severity was established. We showed the presence of ANA in COVID-19, without identifying any particular antibody specificity. ANA are useful biomarkers for the diagnosis and the monitoring of autoimmune rheumatic diseases. Since it has been reported that ANA could precede by several years a symptomatic autoimmune disease^33^, a follow-up of patients could also be advocated.

Interestingly we showed that NETs detected by CitH3 but not exDNA, are associated with severe form of COVID-19. In contrast, only exDNA levels were found significantly higher in severe COVID-19 patients with aCL IgG positivity than in aCL IgG negative ones.

This significant association was not observed for CitH3, possibly because of the low number of patients tested for CitH3. In this line, a major limitation of our study was missing data in CitH3 and exDNA because of insufficient quantity of serum for some patients. However patient characteristics of the exDNA or CitH3 cohorts were comparable to those of the total cohort (Supplementary Table S1), which validate the results. Additionally, the possible difference in origin of exDNA and CitH3 could also explain the difference in results. Nevertheless, our data led us to propose a potential role of aPL in COVID-19: aCL would exacerbate the severity of the disease by affecting exDNA release. ExDNA and other DAMPs molecules, such as CitH3, are major structural elements of NETs. ExDNA release also reflects tissue damage and cell apoptosis. Therefore cells expressing ACE2, the entry receptor for SARS-CoV-2, are potential sources of exDNA during COVID-19 infection. ExDNA represents relevant markers of inflammation^14,15^ and have been proposed in the pathogenesis of inflammatory and autoimmune diseases, such as APS^20^. More recently it has been shown that their levels increased during the evolution of the COVID-19 and thus, exDNA are proposed as biomarkers for the patient outcome^11^. Altogether, our data reinforced the link between exDNA release and auto-immunity in COVID-19.

**Figure 4:**
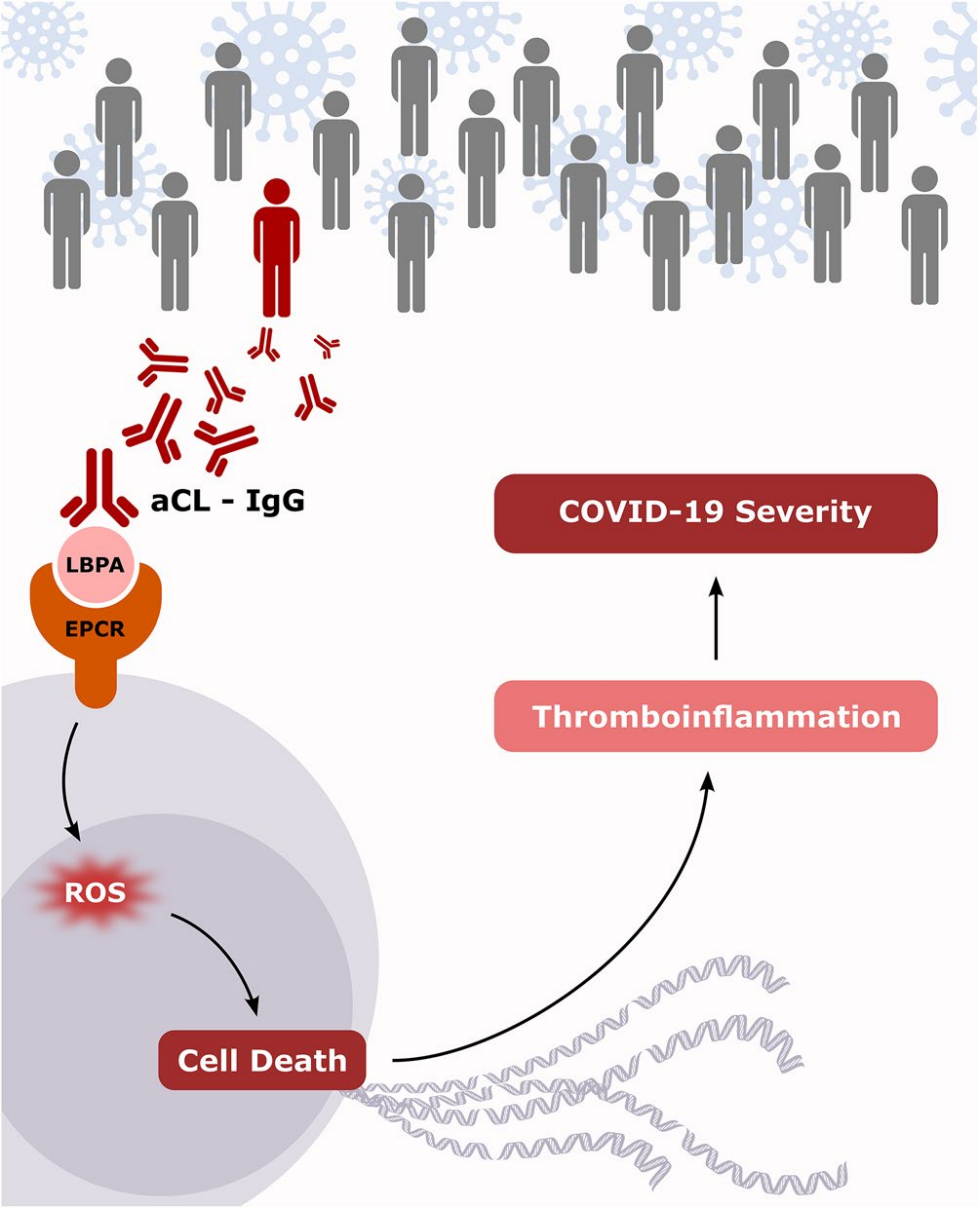
Putative mechanism of anticardiolipin autoantibodies in COVID-19 severity. We hypothesize that SARS-CoV-2 infection of genetically-prone patients results in autoimmunity. Anticardiolipin autoantibodies (aCL) can bind a cell surface complex composed of lysobiphosphatidic acid (LBPA) and endothelial protein C receptor (EPCR). This interaction promotes cell death via reactive oxygen species (ROS). Then, releases extracellular DNA contributes to a heightened thrombo-inflammatory state associated with COVID-19 severity. The figure has been drawn by AB using InkScape 0.92, http://www.inkscape.org.

Pathogenicity of aPL from patients with COVID-19 has also been previously revealed by the fact that injection of IgG fractions isolated from these patients accelerated thrombotic events in an animal model of venous thrombosis, and importantly promoted NET release by human neutrophils^21^. One can speculate that aPL could activate apoptosis of other cell types such as endothelial cells or other cells expressing ACE2.We can thus hypothesize that autoimmunity enhances the deleterious effect of the inflammatory response, through aCL and exDNA release. Since oxidative stress is known to play a critical role in cell death^34^, increase of production of reactive oxygen species (ROS) has been associated with ExDNA release, and we can speculate that ROS formation could be associated with severe damage in COVID-19. In agreement, Wenzhong et al. showed by employing bioinformatics methods that SARS-CoV-2 generates ROS by iron capture and therefore damages the human immune system^35^. The preferential involvement of autoantibodies against cardiolipin in the severity of the disease may be related to a new described mechanism showing that aCL recognize a cell surface complex composed of lysobiphosphatidic acid (LBPA) and endothelial protein C receptor (EPCR)^36^. Subsequent endocytosis activates Toll like receptor 7 and 8 (TLR 7 and TLR8) and type I IFN signaling, leading to further synthesis of autoantibodies and ROS production. As TLR7 and type I IFN signaling are involved in COVID-19, we can hypothesize that aCL generated during the infection could signal through the recently described EPCR-LBPA pathway involved in inflammation and thrombosis. A putative mechanism of aCL role in severe forms of COVID-19 is proposed in Fig. 4.

In conclusion, we propose that severe forms of COVID-19 engage an autoimmune mechanism that may exacerbate inflammatory pathways through exDNA release. Our results have two major applications in the management of COVID-19: the follow-up of patients by the detection of aCL and a proposal of an original therapeutic strategy targeting extracellular DNA.

## Supplementary Information

Supplementary Table S1.

## Data Availability

The datasets used and/or analysed during the current study available from the corresponding author on reasonable request.
