# Supplementary material for: Anti-cardiolipin IgG autoantibodies associate with circulating extracellular DNA in severe COVID-19
Source: Sci Rep. 2022 Jul 22;12:12523. doi: 10.1038/s41598-022-15969-y (PMC9305055; doi:10.1038/s41598-022-15969-y)
Supplement: Supplementary file 1 — Supplementary Table S1. [file 41598_2022_15969_MOESM1_ESM.docx]

**Table 1S**: Characteristics of COVID-19 patients with both exDNA and CitH3 levels available

|  | Mild (N=17) | Severe (N=32) | Total (N=49) | p value |  |
| --- | --- | --- | --- | --- | --- |
| **Age - years** |  |  |  | 0.549^1^ |  |
| Mean (SD) | 67.1 (21.6) | 70.3 (15.5) | 69.2 (17.7) |  |  |
| Range | 20 - 92 | 44 - 96 | 20 - 96 |  |  |
| **Female - n (%)** | 9 (52.9%) | 15 (46.9%) | 24 (49.0%) | 0.682^2^ |  |
| **Duration of Symptoms - days** |  |  |  | **< 0.001^1^** |  |
| Mean (SD) | 7.6 (4.6) | 15.3 (7.8) | 12.7 (7.7) |  |  |
| Range | 1.000 - 14.000 | 4.000 - 31.000 | 1.000 - 31.000 |  |  |
| Missing Values | 1 | 1 | 2 |  |  |
| **Medical History - n (%)** |  |  |  |  |  |
| Thrombosis | 2 (11.8%) | 3 (9.4%) | 5 (10.2%) | 0.793^2^ |  |
| Heart failure | 0 (0.0%) | 2 (6.2%) | 2 (4.1%) | 0.293^2^ |  |
| Stroke | 1 (5.9%) | 3 (9.4%) | 4 (8.2%) | 0.671^2^ |  |
| Coronary heart disease | 2 (11.8%) | 3 (9.4%) | 5 (10.2%) | 0.793^2^ |  |
| Diabetes | 4 (23.5%) | 9 (28.1%) | 13 (26.5%) | 0.729^2^ |  |
| High Blood Pressure | 7 (41.2%) | 18 (56.2%) | 25 (51.0%) | 0.315^2^ |  |
| Chronic renal failure | 3 (17.6%) | 3 (9.4%) | 6 (12.2%) | 0.400^2^ |  |
| Chronic respiratory disease | 1 (5.9%) | 1 (3.1%) | 2 (4.1%) | 0.642^2^ |  |
| Cancer | 3 (17.6%) | 3 (9.4%) | 6 (12.2%) | 0.400^2^ |  |
| **Invasive Ventilation - n (%)** | 0 (0.0%) | 10 (31.2%) | 10 (20.4%) | **0.010^2^** |  |
| **Thrombotic events - n (%)** | 0 (0.0%) | 2 (6.9%) | 2 (5.0%) | 0.372^2^ |  |
| Missing Values | 6 | 3 | 9 |  |  |
| **PN (G/L)** |  |  |  | **0.009^1^** |  |
| Mean (SD) | 4.1 (1.2) | 6.5 (3.6) | 5.7 (3.2) |  |  |
| Range | 2.3 - 7.4 | 2.1 - 15.0 | 2.1 - 15.0 |  |  |
| **Lymphocytes (G/L)** |  |  |  | 0.732^1^ |  |
| Mean (SD) | 1.3 (0.4) | 1.4 (0.8) | 1.4 (0.7) |  |  |
| Range | 0.4 - 1.9 | 0.2 - 3.9 | 0.2 - 3.9 |  |  |
| **NLR** |  |  |  | 0.052 ^1^ |  |
| Mean (SD) | 3.6 (2.2) | 7.3 (7.3) | 6.0 (6.3) |  |  |
| Range | 1.5 - 10.9 | 0.9 - 37.9 | 0.9 - 37.9 |  |  |
| **Eosinopenia** | 7 (41.2%) | 21 (65.6%) | 28 (57.1%) | 0.100^2^ | |
| **Death** | 1 (9.1%) | 6 (21.4%) | 7 (17.9%) | 0.366^2^ | |
| Missing Values | 6 | 4 | 10 |  |  |

**Table 1S legends**: PMN: Polymorphonuclear neutrophils, NLR: Neutrophil-Lymphocyte Ratio, ^1^ Student’s t-test, ^2^ Pearson’s Chi-squared test
